# The natural pattern of birth timing and gestational age in the U.S. compared to England, and the Netherlands

**DOI:** 10.1371/journal.pone.0278856

**Published:** 2023-01-18

**Authors:** Eugene Declercq, Anneke Wolterink, Rachel Rowe, Ank de Jonge, Raymond De Vries, Marianne Nieuwenhuijze, Corine Verhoeven, Neel Shah

**Affiliations:** 1 Community Health Sciences, Boston University School of Public Health, Boston, Massachusetts, United States of America; 2 Amsterdam UMC, Vrije Universiteit Amsterdam, Midwifery Science, AVAG/ Amsterdam Public Health, Amsterdam, The Netherlands; 3 National Perinatal Epidemiology Unit, Nuffield Department of Population Health, University of Oxford, Oxford, England; 4 Center for Bioethics and Social Sciences in Medicine, University of Michigan Medical School, Ann Arbor, Michigan, United States of America; 5 Academie Verloskunde Maastricht, Maastricht, The Netherlands; 6 Department of Obstetrics, Gynecology and Reproductive Biology Harvard Medical School, Boston, Massachusetts, United States of America; National Institute of Public Health: Instituto Nacional de Salud Publica, MEXICO

## Abstract

**Objective:**

To examine cross-national differences in gestational age over time in the U.S. and across three wealthy countries in 2020 as well as examine patterns of birth timing by hour of the day in home and spontaneous vaginal hospital births in the three countries.

**Methods:**

We did a comparative cohort analysis with data on gestational age and the timing of birth from the United States, England and the Netherlands, comparing hospital and home births. For overall gestational age comparisons, we drew on national birth cohorts from the U.S. (1990, 2014 & 2020), the Netherlands (2014 & 2020) and England (2020). Birth timing data was drawn from national data from the U.S. (2014 & 2020), the Netherlands (2014) and from a large representative sample from England (2008–10). We compared timing of births by hour of the day in hospital and home births in all three countries.

**Results:**

The U.S. overall mean gestational age distribution, based on last menstrual period, decreased by more than half a week between 1990 (39.1 weeks) and 2020 (38.5 weeks). The 2020 U.S. gestational age distribution (76% births prior to 40 weeks) was distinct from England (60%) and the Netherlands (56%). The gestational age distribution and timing of home births was comparable in the three countries. Home births peaked in early morning between 2:00 am and 5:00 am. In England and the Netherlands, hospital spontaneous vaginal births showed a generally similar timing pattern to home births. In the U.S., the pattern was reversed with a prolonged peak of spontaneous vaginal hospital births between 8:00 am to 5:00 pm.

**Conclusions:**

The findings suggest organizational priorities can potentially disturb natural patterns of gestation and birth timing with a potential to improve U.S. perinatal outcomes with organizational models that more closely resemble those of England and the Netherlands.

## Introduction

Some industrial countries, most notably the United States, have reportedly experienced a significant shift in the distribution of births by gestational age [1]. Some posit the shift reflects an underlying physiological change [2], while others suggest an increased level of obstetric intervention as the driving force [3, 4]. Recent developments encouraging more widespread use of labor induction at 39 weeks may further shorten the length of gestation [5, 6]. Related to discussions of gestational age distributions are examinations of the timing of births by the hour of day, with similar debates about the extent to which the organization and delivery of care influence variations observed [7].

Studies of the timing of birth date back almost 200 years. As birth registration became more widespread in the twentieth century, studies of birth timing became more feasible, be it by season [8–10], day of the week [11], or hour of the day [12]. Studies of the hour of birth have been done to explore patterns by race and hospital type (private/public) [13]; to identify the relation between birth timing and stillbirths [14, 15]; and to link birth timing to obstetrical interventions [16–20]. Recently, MacFarlane and colleagues, based on data from England and Wales [7, 21], found wide variations based on day of the week, birth setting, and medical interventions. Most prior studies have been based on data from single hospitals, regional collaborations, national hospitals or vital statistics data from a single country [22].

We use data on gestational age and timing of births in home and hospital births in three high income countries with different maternity care systems–the United States, the Netherlands and England–allowing identification of what may be considered natural birth patterns and to speculate on the role that the organization of care might have on the birthing process. While the United States maternity care system primarily relies on obstetricians, the Netherlands and England rely primarily on midwives with obstetric consultants for back-up in higher risk cases [23]. (S1 Table). Notably, the Netherlands and England report perinatal outcomes significantly better than the United States [23]. Our specific objectives were to: (a) describe the change in gestational age distributions in the United States from 1990 to 2020; (b) compare gestational age at birth overall and in home births in the three countries; and (c) describe, explore, and compare the timing of birth by hour of day in hospital settings for vaginal births without induction or augmentation of labour with home births, where such interventions do not occur.

## Methods

### Data

The data for these analyses are drawn from population-based birth data from all three countries, and from studies of birth timing and place of birth done in England [24] and the Netherlands [25]. The numbers of births included in the time of birth analyses for home, all hospital births and hospital vaginal births without induction or augmentation are summarized in S2 Table.

#### United States

The U.S. data are based on approximately 3.7 million annual births drawn from national vital statistics files available through public sources, specifically the public website, CDC Wonder [26], and the public use datasets available for download from the National Center for Health Statistics (NCHS) (https://www.cdc.gov/nchs/data_access/vitalstatsonline.htm). These data come from the National Standard Certificate of a Live Birth, an agreement between individual U.S. states that collect the data, and the National Center for Health Statistics, where agreed upon variables are compiled into a national datafile. A revision of the Standard Certificate in 2003 added hour of birth to the dataset, but the revised certificate was not uniformly adopted; it was not until 2016 that all states included hour of birth in their data collection [27]. The birth timing data are drawn from 2014 for consistency with the Dutch data and include data from 48 states and the District of Columbia, 98.8% of all U.S. births that year. For context, the same data for 2020, involving all states, is presented in S1–S3 Figs.

#### Netherlands

Data from the Netherlands on gestational age were drawn from the 2020 public use datafile of births registered by Perined which includes perinatal data from nearly all of the approximately 165,000 births in that year [28]. Data on hour of the day were from a study based on 2014 Perined data. This national data registry merges data collected from three sources of maternal and perinatal care: primary midwifery care, obstetrician-led care and neonatal care. In 2014, 100% of primary care midwifery practices, 99% of obstetric departments and 88% of neonatal departments submitted their data to Perined [29]. Perined checks the merged dataset for consistency and validates the data through multiple checks [30]. For this study, we used results from an earlier analysis of hour of birth for birthing people who gave birth in the Netherlands in 2014, excluding premature births, multiple gestations, and pre-labour antepartum deaths. In the Netherlands, about 85% of all pregnant people receive primary midwifery care at the onset of pregnancy. If risks factors or complications occur, they are referred to obstetrician-led hospital care [31].

#### England

Data for gestational age of all births in England in 2019 came from the UK Office for National Statistics [32]. Data on hour of birth came from the Birthplace national prospective cohort study which was designed to compare perinatal and maternal outcomes in births planned in different settings in England [24, 33]. Data on 79,774 births were collected between 1^st^ April 2008 and 30^th^ April 2010, and include 32,257 births planned to occur in one of 36 hospital obstetric units that were part of a stratified random sample, 11,666 births planned in 53 freestanding midwifery units (birth centres on a site separate from a hospital obstetric unit), 17,582 births planned in 43 alongside midwifery units (birth centres co-located with a hospital obstetric unit) and 18,269 planned home births from 142 National Health Service (NHS) trusts (organizations providing maternity care). Births were eligible for inclusion if a vaginal birth was planned and some labor care was received from an NHS midwife in her planned birth setting. Those who had an elective caesarean section or caesarean section before the onset of labor, presented in preterm labor (<37 weeks’ gestation), had a multiple pregnancy, an unplanned home birth, or who were “unbooked” (received no antenatal care) were excluded, as were those who had a stillbirth before the start of care in labor. All data were collected on study-specific forms, extracted from the medical records, by midwives attending that labor [33].

### Analysis

Data from the three countries were tabulated by completed weeks of gestation for the gestational age comparisons. Births by hour of day were recorded in 24 hourly increments beginning at midnight and presented as the annual average of these figures. To examine gestational age distributions in the United States from 1990 to 2020, gestational age was determined using last menstrual period, because the currently recommended method, obstetrical estimate related to ultrasound readings, was not available in 1990 [34]. For the examination of U.S. gestational age by place of birth, we used obstetrical estimate to determine gestational age. Gestational age was categorized as < 34, 34–36, 37, 38, 39, 40, 41 and 42+ completed weeks. In the case of England [35, 36] and the Netherlands [37], we used the gestational age reported in their national databases with both countries relying primarily on an ultrasound based estimate.

We compared gestational age distributions for births in the U.S., [26] England [32] and the Netherlands [28] using publicly available data from the respective countries. In the case of home births, we relied on public use datafiles for the Netherlands and U.S., and relied on the Birthplace study [24] for the distribution in England.

We examined birth timing by hour of day in home and hospital births occurring between 37 and 42 completed weeks in the three countries and repeated this analysis for vaginal births in hospitals without induction or augmentation of labour.

#### Regulatory approvals

The Medical Ethics Review Committee of VU University Medical Center confirmed that the Medical Research Involving Human Subjects (WMO) did not apply for the Dutch data and therefore an official approval of the study by this committee was not required (reference number 2020.476). The United States data are publicly available and de-identified. The IRB reviewed the research and made the determination that it was not human subjects research and therefore had no requirement to obtain consent. The study from which the English data were drawn was given approval by the Berkshire Research Ethics Committee in October 2007 (reference number: 07/H0505/151), with an amendment to the original protocol approved by a sub-committee in April 2008. All the data collected were routinely recorded in the maternity, postnatal or neonatal notes and did not include personally identifiable data.

## Results

### Gestational age at birth

**Fig 1:**
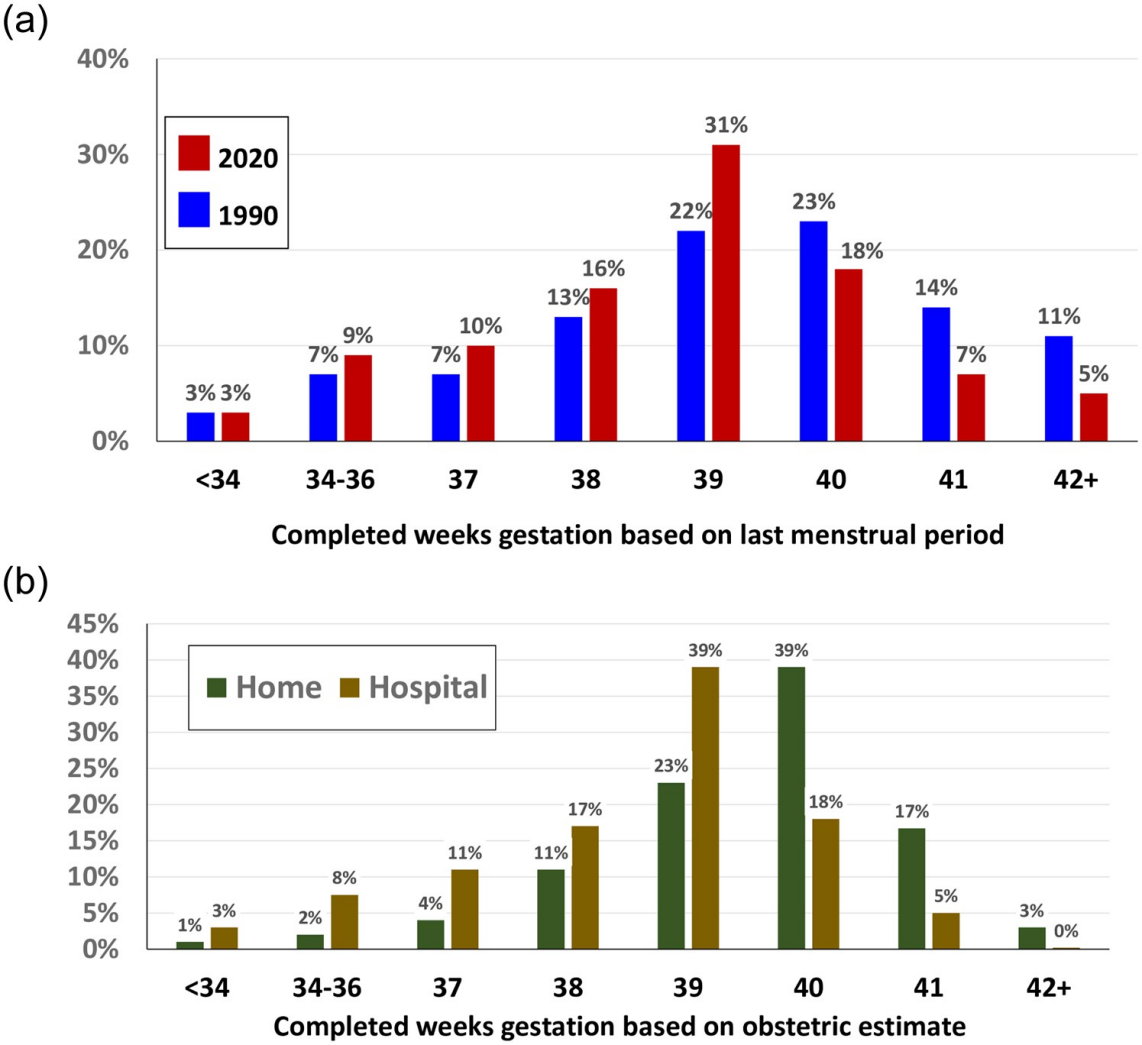
a. Gestational Age, U. S., All Births, 1990, 2020. Sources: U.S.: National Vital Statistics System, 1990 & 2020 Public Use Datasets. Note: While NCHS recommends use obstetrical estimate of gestational age, it was not available in 1990, thus comparison is based on last menstrual period for consistency between 1990 and 2020 measure. b. Gestational Age Distribution by Place of Birth, U. S., 2020. Source: National Vital Statistics System. 2020 Public Use Dataset.

There was a notable shift in the U.S. distribution of gestational age at birth, based on last menstrual period, over the 30-year period between 1990 and 2020 (Fig 1a). In 1990, almost half of all U.S. births (48%) occurred at ≥40 weeks, with 40 weeks being the modal gestational age. By 2020, less than a third (30%) of births occurred at ≥40 weeks, with 39 weeks being the most common gestational age. The mean gestational age in 1990 was 39.1 weeks (S.D. 2.7), while in 2020, using the last menstrual period measure, it was 38.5 weeks (S.D. 2.5). Over the same period, there was only minor change in the gestational age distribution of home births in the United States. In 1990 there was no difference in the mean gestational age (39.1 weeks) between home and hospital births in the U.S., while by 2020, the difference was a full week: 39.5 weeks at home; 38.5 in hospitals.

Fig 1b presents gestational age distributions for 2020 U.S. births by place of birth based on the now standard obstetrical estimate of gestational age [34]. There was a peak of home births at 40 weeks (39% of all births), with 59% at ≥40 weeks and a mean gestational age of 40.2 weeks (S.D. 7.2). Hospital births had a mean gestation of 38.4 weeks (S.D. 2.4), and only 23% occurred at ≥40 weeks, with the highest proportion (39%) occurring at 39 weeks.

**Fig 2:**
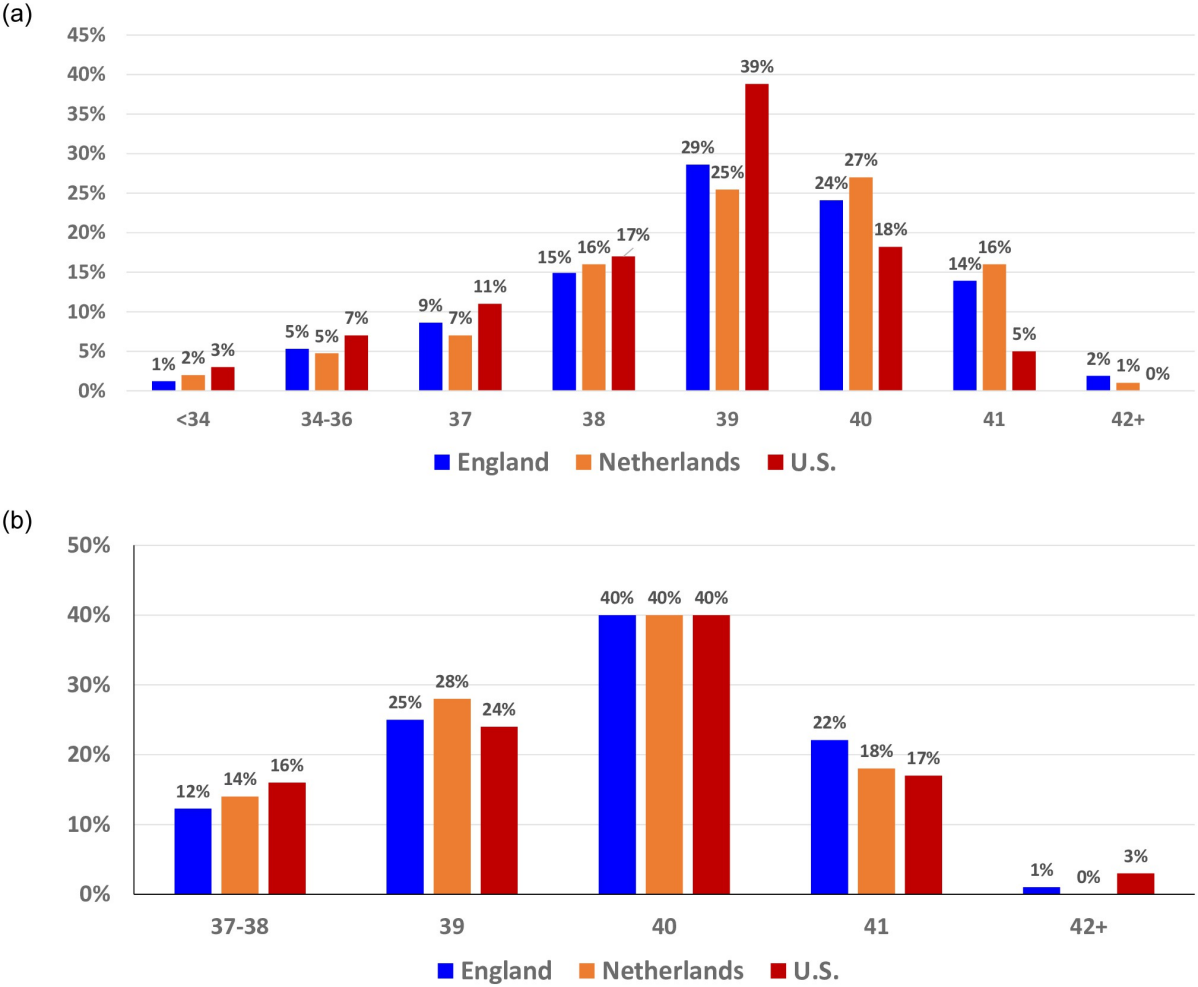
a. 2020 Gestational Age Distributions, All Births, England, Netherlands and U.S. Source: U.S.: CDC Wonder. England: Office for National Statistics. Netherlands: Peristat.nl. b. Gestational Age Distribution* Home Births, England, 2008–2010 & Netherlands & U.S. 2020. * To compare to data from England, data limited to births at 37+ weeks; “0%” refers to < 0.5%. Sources: U.S.: CDC Wonder. England: Birthplace Study. Netherlands: Peristat.nl.

Fig 2a, drawn from 2020 data from public databases in the respective countries, once again shows the distinct peak of the U.S. at 39 weeks compared with England (29%) and the Netherlands (25%). In the U.S., three-fourths of births (76%) occurred before 40 weeks, compared to the Netherlands at 56% and England at 60%. Fig 2b, limits the comparison to home births using 2020 data for the U.S. and Netherlands, and Birthplace study data (2008–10) for England. Because the English study was limited to full term births, we limited the U.S. and Dutch data to births at 37+ weeks gestation. It shows a similar pattern in each country with a clear peak of 40% of births at 40 weeks in all three countries.

### Births by time of day

**Fig 3:**
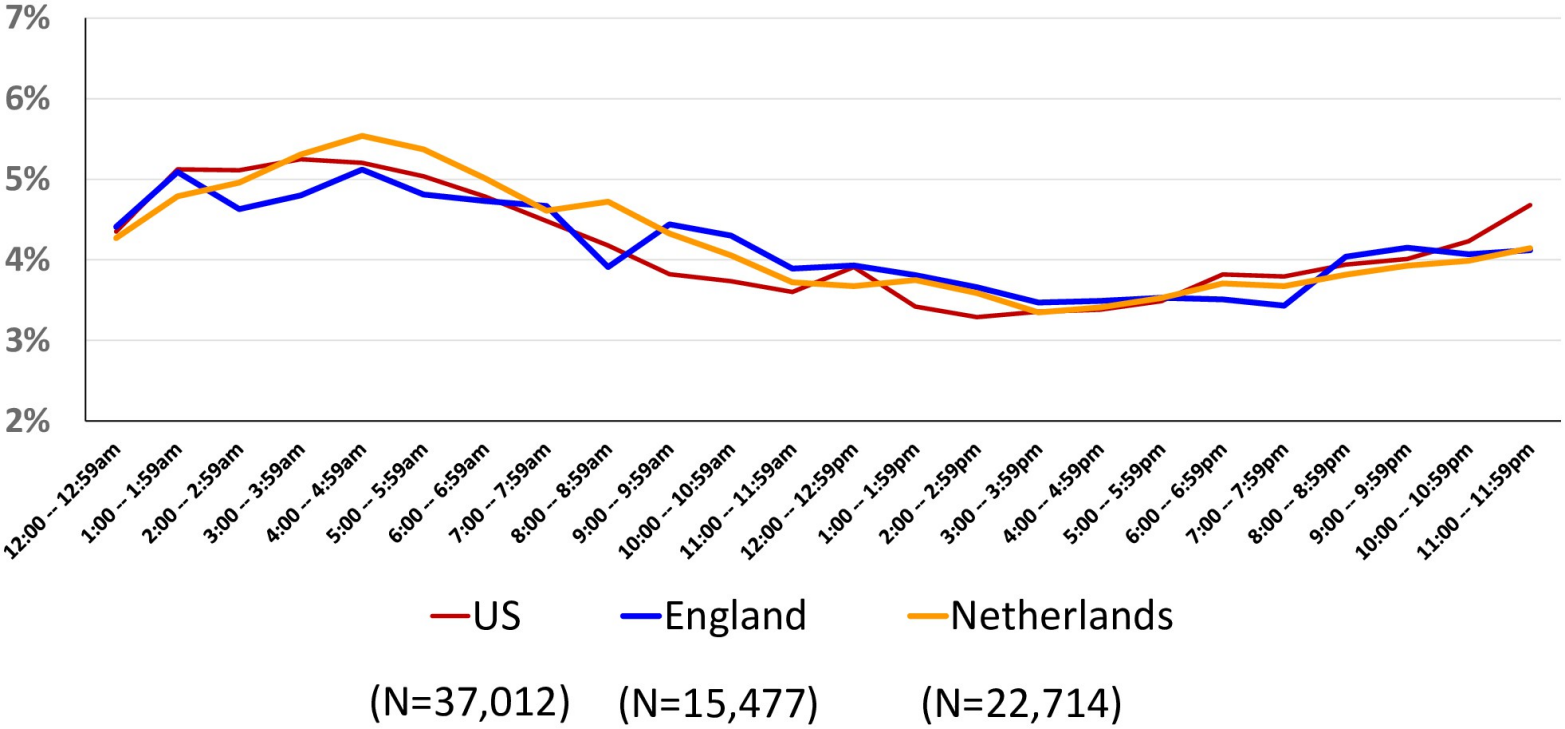
Home births by hour of day, U.S. & Netherlands, 2014 & England 2008–10. Source: U.S.: National Vital Statistics System, 2014 Public Use Dataset; England: Birthplace Study; Netherlands: Peristat.nl.

Fig 3 displays the pattern of home births in the three countries by hour of the day. Because the English sample excluded births prior to 37 weeks, we made the same exclusion for the U.S. and the Netherlands. The pattern is similar across all three settings, with a peak in the early morning hours, roughly between 1:00am and 5:59am, followed by a steady decline bottoming out between roughly 2:00pm and 4:59pm.

**Fig 4:**
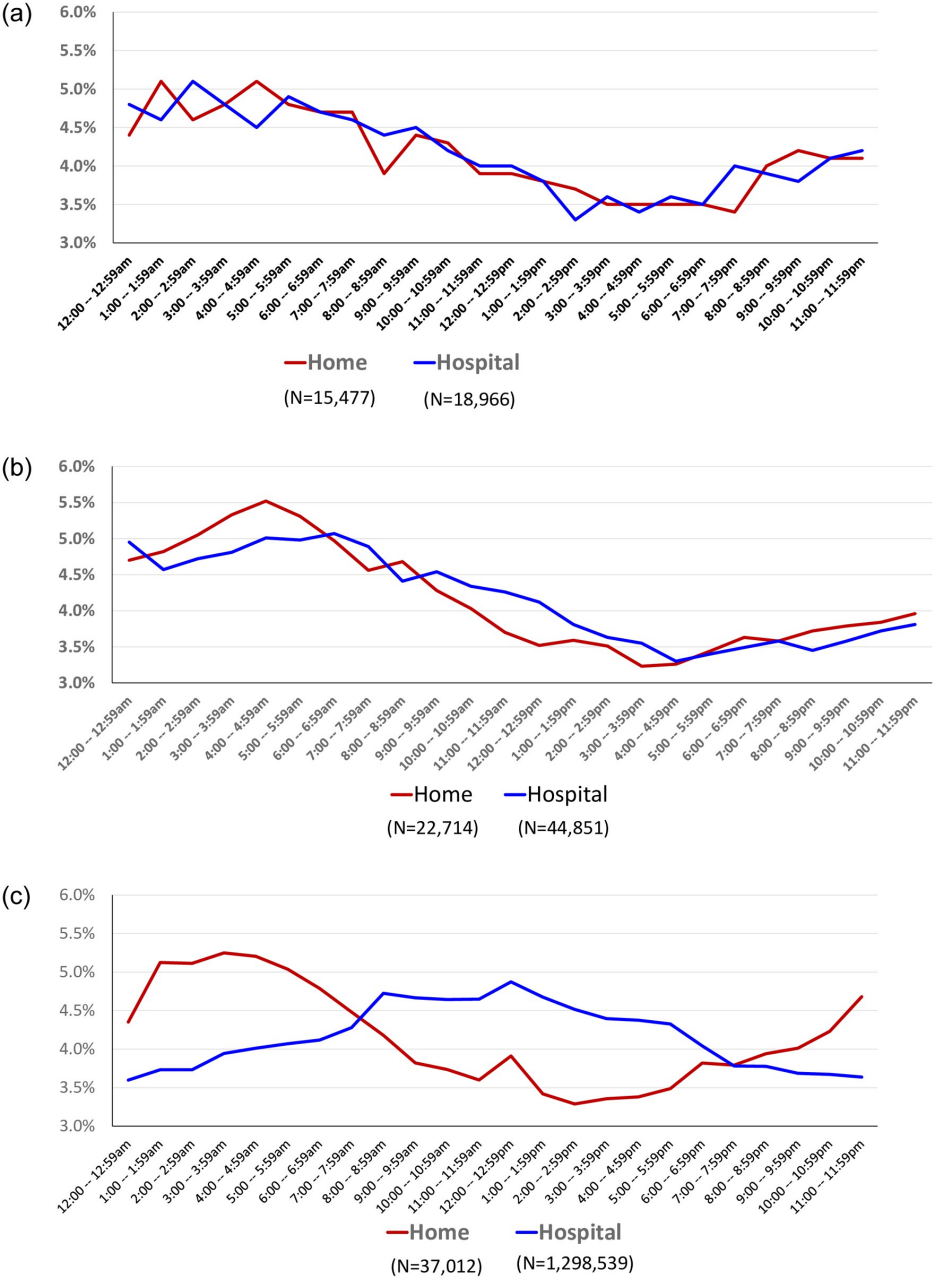
a. Births by Hour of Day and Place of Birth, (Home & Hospital Vaginal no Induction or Augmentation), England 2008–2010. Source: Birthplace Study [24]. b. Births by Hour of Day and Place of Birth, (Home & Hospital Vaginal no Induction or Augmentation), Netherlands, 2014. Source: Peristat.nl. c. Births by Hour of Day and Place of Birth, (Home & Hospital Vaginal no Induction or Augmentation), U.S. 2014. Source: National Vital Statistics System 2014 Public Use Dataset.

Fig 4a through Fig 4c present comparisons for England, the Netherlands and the U.S. between the timing of home births and hospital vaginal births without induction or augmentation [38]. In Fig 4a, the time of birth in England varies little between home and hospital. Likewise in the Netherlands (Fig 4b) the patterns are largely similar, though home births show a higher early morning (4:00–4:59 am) peak. In the case of the U.S. (Fig 4c) however, the patterns in home and hospital births are inverse, with a hospital peak conforming roughly to traditional business hours (7:00am-5:00pm), even in the case of vaginal births with no induction or augmentation–interventions that would likely alter the timing pattern. Concern that the 2014 pattern in the U.S. was unique is addressed by the comparison of data from 2014 and 2020 found in the S1 Fig, showing that these distinct patterns have remained consistent over time. S2 Fig compares the timing of U.S. home births in 2014 and 2020 and birth center births in 2020. The timing of home births changes little over the 6 year period. Births in freestanding birth centers display a comparable pattern, though with a higher rate in the early evening. S3 Fig also includes US data for 2020 on timing of hospital vaginal births with no induction or augmentation and also without reported anesthesia medication. In that case the pattern was slightly more similar to the home birth pattern, with a peak between 5:00 am and 8:59 am.

## Discussion

### Main findings

We found that over the course of 30 years, average gestational age in the U.S. was reduced by more than one half week. To examine the possible impact of the organization of maternity care on gestational age at birth, we compared home and hospital births in the U.S. with comparable births in two countries with different organizational models of maternity care: the Netherlands and England. We found that overall, the U.S. obstetrics-centered model had a distinctly larger proportion of all births at ≤39 weeks compared with the midwifery-centered models of England and the Netherlands. Gestational age distributions for home births in the U.S., the Netherlands and England, however, were very similar, with a modal category of 40 weeks.

We next examined the pattern of births by time of day. In all three countries we found that home births had strikingly similar diurnal patterns in the timing of birth, with a clear peak in the hours from 1:00am to 5:59am.

Limiting the hospital births to vaginal births without interventions that could alter the timing patterns (inductions or augmentation) brought the U.S. differences into sharp relief. In this restricted sample, the timing of births at home and in hospital in England and the Netherlands was largely the same. In the U.S., however, a decidedly different pattern emerged. Vaginal births without induction or augmentation in U.S. hospitals were far more likely to occur during standard working hours.

A full explanation of the reasons for this difference is beyond the scope of this study and merits future research. The undercounting of induction [39] and augmentation [40] in U.S. birth certificate data may contribute to those differences. Possible explanations also include a greater emphasis on active management of labor and ample access to easily administered Pitocin [41] and the potential impact of anesthesia administration, given the finding that the pattern of U.S. births without anesthesia conformed more closely in timing to home births, combined with the high rate of epidural use in vaginal births (74% in 2020) in the U.S. [26].

The consistency of the data on the timing of home births strongly suggests a natural pattern whereby birth without intervention is more likely to occur in the early hours of the morning. That this is the natural pattern of the timing of ‘undisturbed’ birth is consistent with studies of the timing of births dating from earlier in the twentieth century when there was less medical intervention in the management of labor [42–44].

For the purposes of hospital organization, the allocation of human resources, and provider and support staff convenience, the timing of birth, at least in the United States, appears to have evolved into a pattern that is easier for the maternity care system to manage. A variety of studies of birth outcomes reference an “evening effect” and “weekend effect.” Some of these studies document poorer outcomes during evening and late night hours for both infants [45–48] and birthing people [49], as well as poorer outcomes on weekends [50], with organizational limitations in providing care during the night often seen as a contributing factor.

The organization and culture of maternity care practice in a given hospital can have a profound impact on the nature of care provided, including the likelihood and timing of a cesarean section [18, 51–54]. For example, in the Netherlands differences between provinces were found in the rates of medical interventions that could not be explained by maternal factors, most notably in type of medical pain relief and referral to a neonatologist [55]. That study also found that higher rates of medical interventions were not associated with better maternal or neonatal outcomes. This finding calls into question the necessity of medical interventions and the influence of organizational culture in decision making. Similarly, time of day may influence the decision to perform medical interventions [18].

### Strengths and limitations

To our knowledge, this is the first international study using large datasets to compare gestational age and timing of birth between three high income countries. Nevertheless, this study is not without limitations. Any study drawing on data from different time periods and different countries will face limitations based on comparability of measures [56, 57]. Wherever possible, we used data that allowed us to create comparable categories. For example, since the 1990 U.S. data used a measure of gestational age based on last menstrual period, we used that measure again in 2020, in spite of the fact that current practice recommends use of obstetrical estimation [58]. Furthermore, we were unable to use data from the same years across countries. In terms of birth timing, the English data was drawn from a 2008–2010 study, while the Dutch data used in the analyses reported here was from 2014 [25]. We had more control over the U.S. data and used the 2014 period for comparison, although the slow adoption by U.S. states of the revised National Certificate of a Live Birth–which added time of birth–meant that U.S. data from 2014 did not include two states accounting for 1.2% of U.S. births, Connecticut and Rhode Island [27]. However, when we repeated our analyses using data from 2020, we found no differences from our analyses of 2014 data (S1 Fig). The data from the US and the Netherlands were from routinely collected national databases, whereas data from England were collected in a national prospective cohort study that may be more susceptible to biases including, for example, selection bias. The exclusion of higher risk births as well as planned cesareans may explain why the timing of spontaneous vaginal hospital births in England we found varied somewhat from a recent population based study from England [59]. However, the study in which the data were collected had a high response rate, and data were collected without requiring consent from the birthing person, minimizing the biases commonly associated with this type of study [33].

Another limitation of comparing home and hospital births is the difference in pregnancy risk factors and complications, although it is unlikely that these may influence the average timing of spontaneous onset of labor [31]. Studies of the reliability of the U.S. birth certificate data on reporting key variables here finds high accuracy in terms of gestational age and method of delivery and a tendency to undercount inductions and augmentation, which may help explain the unique timing pattern of U.S. hospital spontaneous vaginal births [40]. Those giving birth at home also differ in important ways (e.g. parity) from those choosing to give birth in hospitals [60], which may also account for some of the home vs hospital differences in timing. Population differences across countries heightens the importance of the striking consistency in the timing of home births across three countries. The large differences between gestational ages among home and hospital births are likely to be the effect of the high rate of medical interventions among hospital births, which contributes to iatrogenic preterm births [61]. Although fewer medical interventions occur among home births, we found slightly lower gestational ages for home births in the Netherlands.

Future research on this topic can help resolve some of the questions raised in this paper. Specifically, comparative, prospective studies that simultaneously explored not only birth timing in home and hospital births, but also hospital maternity care practices could better identify differences that might impact timing and outcomes. The recent concern in England that an emphasis on “normal birth” is negatively impacting outcomes at a time when the cesarean rate in England is rapidly rising [62] suggest the need for a more comprehensive understanding of nature of maternity care practice. There is also a need for better documentation of induction and augmentation in hospital births to assess what impact, if any, they have on birth timing. Further research could also clarify whether the practice patterns of some Dutch midwives (e.g. sweeping membranes to start labor) influences timing of birth in the larger population [63].

## Conclusion

If there is a medical reason for offering an intervention, it may be beneficial to consider the natural pattern of labour in planning that intervention. More intentional examination of the organizational model of maternity care in the United States may benefit from lessons in other industries (e.g. criminal justice) where resources and operations were adjusted to correspond to need rather than organizational convenience [64, 65].

England and the Netherlands, both of which have better infant and maternal outcomes than the U.S., rely heavily on midwives to provide maternity care, which may reflect both a difference in the allocation of resources and a preference for spontaneous labor over intervention [23]. The fact that spontaneous non-intervened vaginal hospital births in England and the Netherlands exhibit a temporal pattern very similar to home births raises the question of whether U.S. maternity care outcomes might improve from a similar practice.

The positive outcomes associated with births in freestanding birthing centers–where, unlike lower risk hospital births in the U.S., the timing pattern is similar to home births (S2 Fig)–and, like home births, the primary attendant is a midwife [66], suggests that there may be an advantage to letting labor take its own course and adapting our institutions to that pattern [67–70]. The question raised by this study is simple: would U.S. maternity outcomes improve if care were organized to correspond to the natural rhythms of labor and the needs of laboring persons, rather than organizational imperatives?

## Supporting information

S1 Table Comparative contextual and perinatal outcome data, Netherlands, U.K. and U.S. (DOCX)

S2 Table Place of birth comparative data from England, the Netherlands and the U.S. (DOCX)

S1 Fig Timing of U.S. Hospital births 2014 and 2020. (TIF)

S2 Fig Timing of U.S. home births 2014 and home and birth center births 2020. (TIF)

S3 Fig Births by hour of day (Home & Hospital Vaginal no Induction/Augmentation /Anesthesia), U.S. 2020. (TIF)
