# Supplementary material for: The natural pattern of birth timing and gestational age in the U.S. compared to England, and the Netherlands
Source: PLoS One. 2023 Jan 18;18(1):e0278856. doi: 10.1371/journal.pone.0278856 (PMC9847908; doi:10.1371/journal.pone.0278856)
Supplement: S1 Table — (DOCX) [file pone.0278856.s001.docx]

**Table S1 Comparative Contextual and Perinatal Outcome Data, Netherlands, U.K. and U.S.**

|  | **Year** | **Netherlands** | **U.K.** | **U.S.** |
| --- | --- | --- | --- | --- |
| Live births (000) | 2019 | 169.7 | 712.6 | 3,747.5 |
| Crude birth rate ^(29)^ | 2019 | 9.8 | 10.7 | 11.4 |
| GDP per capita US$ | 2020 | 57,719 | 43,882 | 62,941 |
| Infant Mortality (per 1,000 live births) | 2019 | 3.6 | 3.7 | 5.7 |
| Perinatal Mortality (per 1,000 live births) | 2018 | 4.9 | 6.2 | 5.8 |
| Maternal Mortality (per 100,000 live births) | 2019 | 5.3 | ^a^6.5 | 20.1 |
| % Low birth weight | 2019 | 5.7 | 6.8 | 8.3 |
| % Cesarean births | 2019 | 15.7 | 29.5 | 31.7 |
| % Births delivered by MD^b^ | 2017 | 39.1 | 30.0 | 90.0 |
| % Births delivered by Midwives^b^ | 2017 | 51.3 | 70.0 | 10.0 |
| % Births at home | 2020 | 14.6 | ^c^2.3 | 1.3 |
| Midwives licensed per 1,000 births | 2019 | 25.4 | 52.3 | ^d^4.0 |
| Obstetricians per 1,000 births | 2019 | 9.8 | 11.6 | ^e^11.6 |

Source: Unless otherwise noted – OECD Health Data, 2022

1. 2017
2. Source: Kennedy, H. The role of midwifery and other international insights for maternity care in the United States: An analysis of four countries. *Birth* 2020;47:332-345.
3. England and Wales 2019
4. 2016
5. 2018
