# Supplementary material for: The natural pattern of birth timing and gestational age in the U.S. compared to England, and the Netherlands
Source: PLoS One. 2023 Jan 18;18(1):e0278856. doi: 10.1371/journal.pone.0278856 (PMC9847908; doi:10.1371/journal.pone.0278856)
Supplement: S2 Table — (DOCX) [file pone.0278856.s002.docx]

**Table S2. Place of birth comparative data from England, the Netherlands and the U.S.**

|  | Home | Hospital (All) | Hospital  (vaginal births with no induction or augmentation)  % hospital Births (N) |
| --- | --- | --- | --- |
| England (2008-2010) | 15,477 | 40,623 | 46.8% (18,996) |
| Netherlands (2014) | 22,714 | 133,558 | 33.6% (44,851) |
| U.S. (2014) | 34,731 | 3,794,692 | 31.3% (1,187,625) |
