# Supplementary figures and images for: The natural pattern of birth timing and gestational age in the U.S. compared to England, and the Netherlands
Source: PLoS One. 2023 Jan 18;18(1):e0278856. doi: 10.1371/journal.pone.0278856 (PMC9847908; doi:10.1371/journal.pone.0278856)

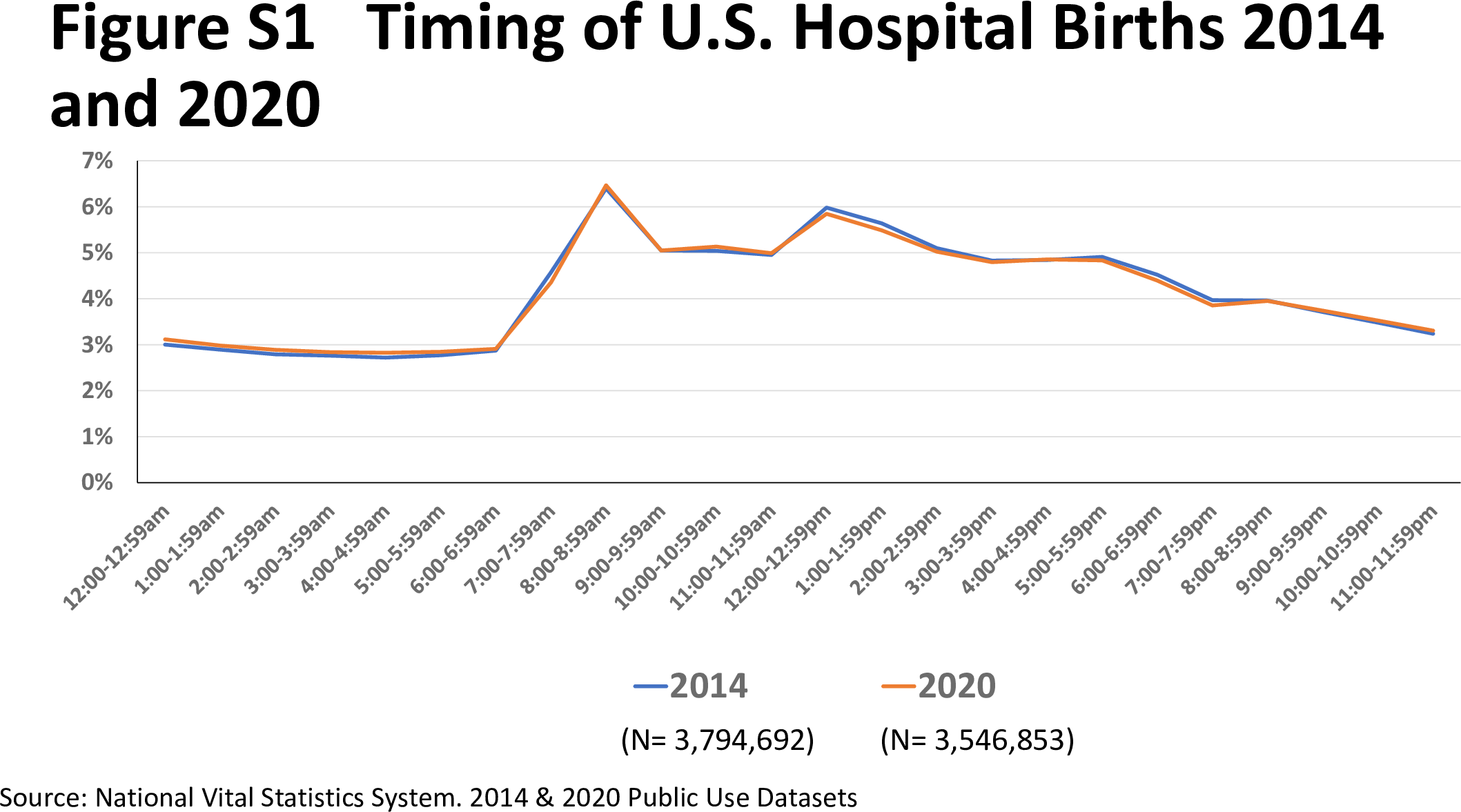

Supplement: S1 Fig — (TIF) [file pone.0278856.s003.tif]

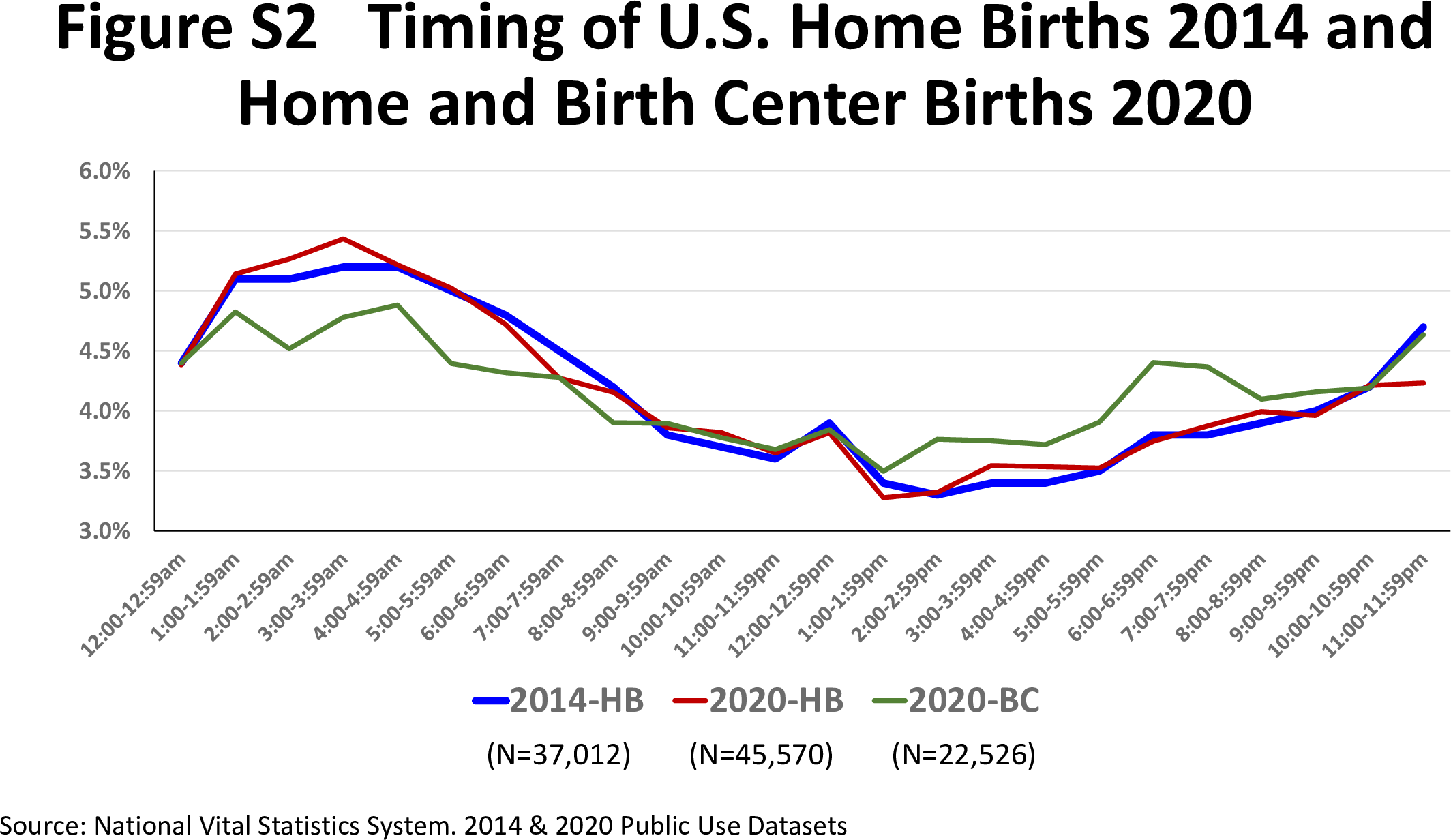

Supplement: S2 Fig — (TIF) [file pone.0278856.s004.tif]

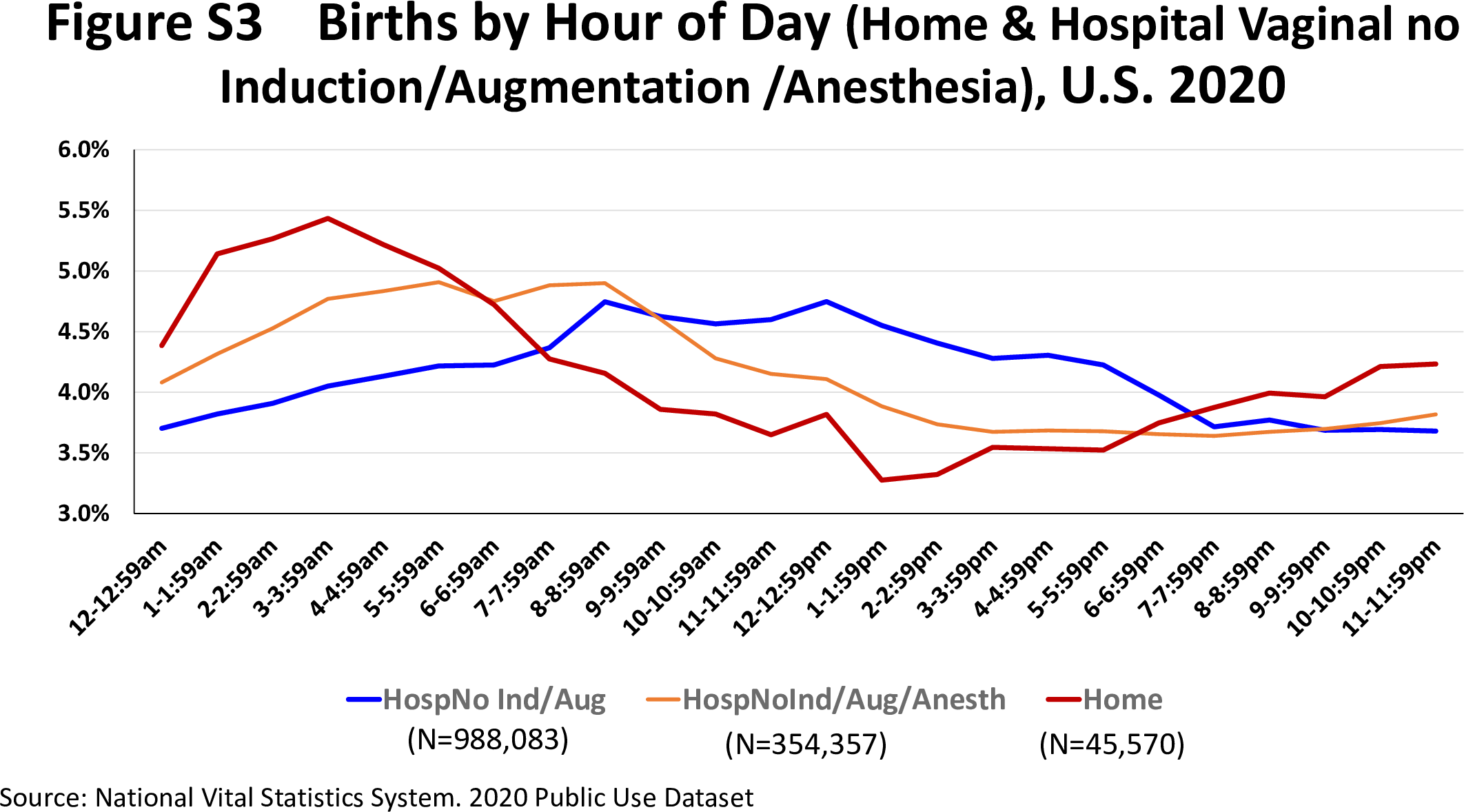

Supplement: S3 Fig — (TIF) [file pone.0278856.s005.tif]
